# The regulatory role of AP-2β in monoaminergic neurotransmitter systems: insights on its signalling pathway, linked disorders and theragnostic potential

**DOI:** 10.1186/s13578-022-00891-7

**Published:** 2022-09-08

**Authors:** Mohamed H. Al-Sabri, Maryam Nikpour, Laura E. Clemensson, Misty M. Attwood, Michael J. Williams, Mathias Rask-Anderson, Jessica Mwinyi, Helgi B. Schiöth

**Affiliations:** 1 Department of Surgical Sciences, Division of Functional Pharmacology and Neuroscience, Biomedical Center (BMC), Uppsala University, Husargatan 3, P.O. Box 593, 75124 Uppsala, Sweden; 2 Department of Immunology, Uppsala University, Dag Hammarskjölds väg 20, 751 85 Uppsala, Sweden; 3 Present Address: Department of Medical Sciences, Uppsala University, BMC, Husargatan 3, 750 03 Uppsala, Sweden

**Keywords:** Transcription factor AP-2 beta, AP-2β, *TFAP2Β*, Monoaminergic neurotransmitter systems, Dopamine, Noradrenaline, Serotonin, Monoamine neurotransmitter disorders, Polymorphisms, Obesity, Neuroblastoma, Diagnostic biomarker and therapeutic target

## Abstract

Monoaminergic neurotransmitter systems play a central role in neuronal function and behaviour. Dysregulation of these systems gives rise to neuropsychiatric and neurodegenerative disorders with high prevalence and societal burden, collectively termed monoamine neurotransmitter disorders (MNDs). Despite extensive research, the transcriptional regulation of monoaminergic neurotransmitter systems is not fully explored. Interestingly, certain drugs that act on these systems have been shown to modulate central levels of the transcription factor AP-2 beta (AP-2β, gene: TFAP2Β). AP-2β regulates multiple key genes within these systems and thereby its levels correlate with monoamine neurotransmitters measures; yet, its signalling pathways are not well understood. Moreover, although dysregulation of TFAP2Β has been associated with MNDs, the underlying mechanisms for these associations remain elusive. In this context, this review addresses AP-2β, considering its basic structural aspects, regulation and signalling pathways in the controlling of monoaminergic neurotransmitter systems, and possible mechanisms underpinning associated MNDS. It also underscores the significance of AP-2β as a potential diagnostic biomarker and its potential and limitations as a therapeutic target for specific MNDs as well as possible pharmaceutical interventions for targeting it. In essence, this review emphasizes the role of AP-2β as a key regulator of the monoaminergic neurotransmitter systems and its importance for understanding the pathogenesis and improving the management of MNDs.

## Background

The monoaminergic neurotransmitter systems, including dopaminergic (DA), adrenergic, noradrenergic (NA) and serotonergic (5-HT) circuitries, regulate a wide range of neurological functions. Dysregulation of these systems is associated with a variety of neuropsychiatric, neurodevelopmental and neurodegenerative disorders, which forms the basis for the so-called monoamine neurotransmitter disorders (MNDs) [1, 2], including obesity, type 2 diabetes (T2D), anxiety, depression, alcoholism, Alzheimer's disease (AD), and neuroblastoma, several of which are among the leading causes of death and disability worldwide [3].

The intricate interplay between monoamine neurotransmitters in the pathophysiology of MNDs poses a substantial challenge for treatment strategies. For instance, dopamine and serotonin, together with norepinephrine, regulate neurological functions ranging from eating behaviour and memory to psychiatric disorders, including aggression, anxiety and depression [4–10]. On the other hand, current treatment options targeting different proteins in monoaminergic systems are far from optimal. In particular, tricyclic antidepressants (TCA), selective serotonin reuptake inhibitors (SSRIs), serotonin and norepinephrine reuptake inhibitors (SNRIs), monoamine oxidase inhibitors (MAOIs) and catechol-*O*-methyltransferase inhibitors (COMTIs) are associated with serious adverse and off-target effects as well as interactions with foods and other drugs, resulting in poor patient compliance and treatment outcomes [11–18].

The transcription factor activating protein 2 beta (AP-2β, gene: *TFAP2Β*) AP-2β has emerged as a vital transcription factor, regulating multiple key genes in monoaminergic neurotransmitter systems such as the serotonin transporter (*5-HTT*) [19, 20], *COMT* [21], dopamine-beta-hydroxylase (*DBH*) [22], vesicular monoamine transporter 2 (*VMAT2*) [23] and others. Subsequently, AP-2β levels correlate positively with monoamine neurotransmitter indices in the brain [24, 25]. In terms of MNDs, not only is *TFAP2Β* associated with reduced anxiety [26, 27], alcoholism [28], obesity [29], binge-eating disorder (BED) [30]and T2D [31], but it also plays a neuroprotective role in AD [32] and neuroblastoma [33]. Together with its role in MNDs, the ability of AP-2β to selectively regulate key genes in the monoamine neurotransmitter pathways underline its potential in the early diagnosis and management of associated MNDs [33–37]. This is supported by the fact that certain drugs acting on monoaminergic neurotransmitter systems, phenelzine (MAOI) and citalopram (SNRI), have been shown to modulate the brainstem levels of AP-2β [38, 39]. Therefore, in order to improve current treatment options for MNDs, a better understanding of AP-2β role in the regulation of monoaminergic neurotransmitter systems is essentially required.

Despite extensive studies on AP-2β crucial role in monoaminergic neurotransmitter systems, little is known about its signalling pathways in controlling these systems, making the underlying mechanisms for associated MNDs rather ambiguous. Thus, we review studies on the AP-2β basic structure, its overarching signalling pathways in the control of monoaminergic neurotransmitters and its polymorphisms associated with MNDs. Also, we discuss possible underlying mechanisms for these associations. Most importantly, we explore its usefulness as a diagnostic biomarker for specific MNDs and underline key opportunities and challenges for targeting it.

### The AP-2 transcription factor family, structure and transduction mechanisms

**Fig. 1:**
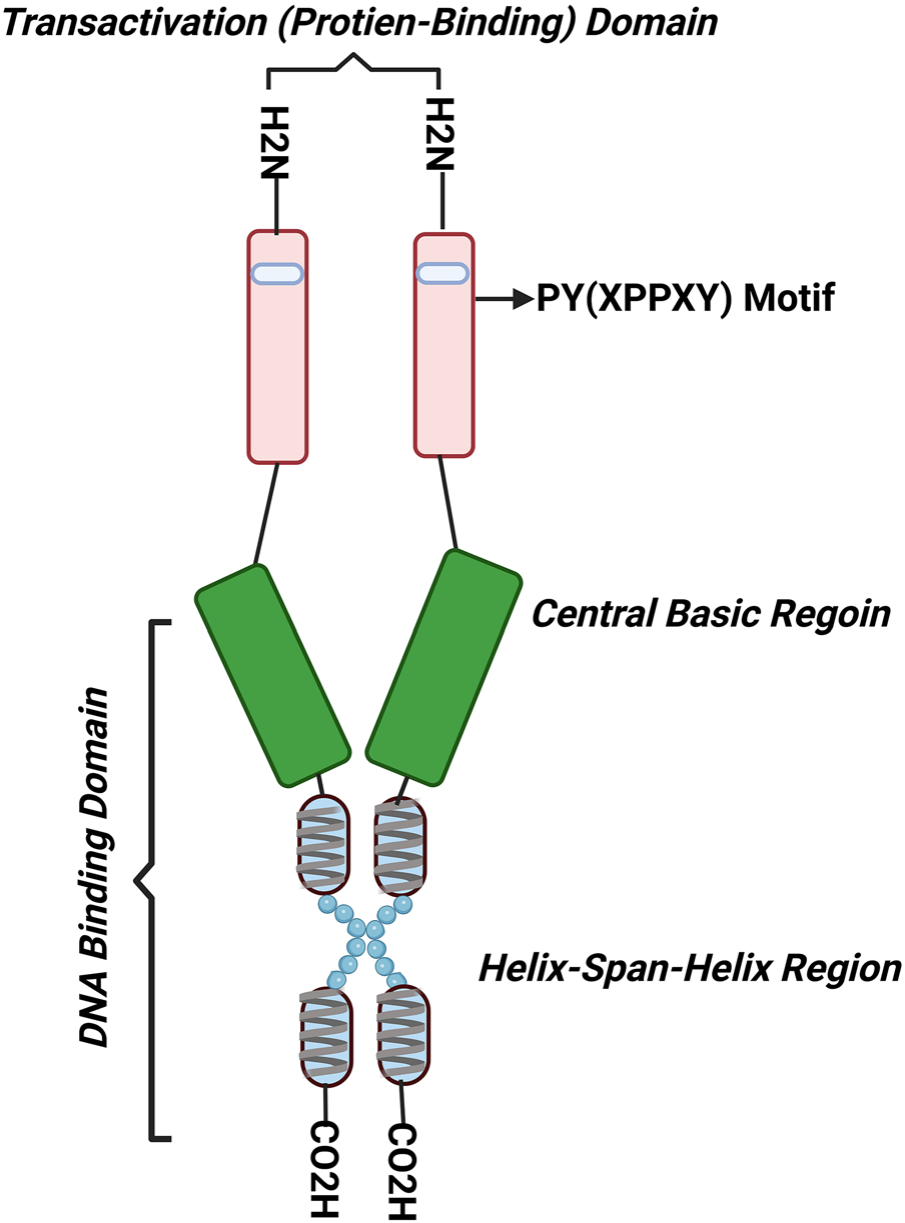
Schematic representation of the possible structure of AP-2β. DNA binding domain is comprised of helix-span-helix and a central basic region, whereas the protein binding domain, also known as the transactivation region, has a special PY motif that is highly conserved. X denotes any amino acid. The picture was influenced by [42, 44, 45, 54], and SwissProt ID: Q92481 & Q61313 and created with Biorender.com

The transcription factor activating protein 2 (AP-2) was first cloned in 1987 [40]. At present, five members of the transcription factor AP-2 have been identified: AP-2α, AP-2β, AP-2γ, AP-2δ and AP-2ε, which are encoded by *TFAP2A/α, TFAP2Β/β, TFAP2C/γ, TFAP2D/δ, TFAP2E/ε* respectively [41]. The AP-2 proteins are highly conserved across species [42] and have differential expression and functions, thereby their mutations give rise to diverse disorders, see Table 1. Structurally, they can form either hetero- or homodimers with a molecular weight of around 50 kDa. They share three regions: a highly conserved C-terminal helix–span–helix homodimerization motif, which starts with a glutamine amino acid; followed by a central basic region, both regions constitute the DNA binding domain; and a less conserved N-terminal proline- and glutamine-rich transactivation region responsible for protein binding domain [42–45] (Fig. 1).

**Table 1:** Expression, function, and linked diseases and disorders for transcription factors AP-2

AP-2 Name	Expression	Function	Linked diseases and disorders
AP-2α/ *TFAP2A*	Neural crest cells & tube of CNS PNS including facial and limb mesenchyme, extraembryonic trophectoderm, human villous cytotrophoblast cells, breast, skin, kidney, retina, adipose tissue, and bone. cartilage and others [42, 61, 242–246]	Regulation of monoamine turnover [62] Specification of GABAergic and glycinergic interneurons [63] Regulate melanocyte [247] and nephron differentiation [248] Play a key role in trophectoderm development [249] A suppressor of chondrocyte differentiation during cartilage development [243, 245, 246]	Mutation causes Branchio-oculo-facial syndrome (BOFS) and ectopic thymus; anophthalmia-microphthalmia syndrome [115, 250] As an activator of gallbladder carcinoma [251] As a suppressor of hepatocellular carcinoma, breast cancer, glioblastoma, melanoma, gastric, prostate, and colorectal cancers[115, 252–257] As an oncogene in acute myeloid leukaemia, squamous cell, nasopharyngeal and pancreatic cancers and neuroblastoma [115, 258–262]
AP-2β/ *TFAP2B*	Fatal neural crest cells, sympathetic neuroblasts of CNS and PNS including facial and limb mesenchyme Heart, smooth and skeletal muscles, collecting duct and distal tubules, kidney, parts of the reproductive system such as prostate and endometrium, retina, adipose tissue, respiratory and endocrine systems including thyroid, adrenal medulla, mammary, sweat, salivary glands and skin [34, 42, 46, 58, 61, 80, 82, 214, 216, 242]	Enhance monoaminergic neurotransmitter activity including serotonergic, dopaminergic and adrenergic transmission through controlling the key genes in monoaminergic neurotransmitter systems [24, 30, 46, 62, 82, 149] Crucial for intrauterine growth, differentiation of distal nephrons and the sympathetic ganglia and neurons, maturation of chromaffin cells of the adrenal medulla and specification of GABAergic and glycinergic interneurons[46, 46, 63, 80] Regulation of adipocytokines, insulin signalling and fat metabolism[29, 34, 134, 135, 263] Proliferation and differentiation of extraembryonic trophectodermal cells [244, 264]	Mutation causes Char syndrome and Patent ductus arteriosus 2, dental anomalies, and terminal renal failure [64–67, 265] Unfavourable prognostic factor in neuroblastoma[33], lung adenocarcinoma[216], breast [221–223] & thyroid cancers [215] Favourable prognostic factor in endometrial carcinoma [217], cervical cancer [224, 225], & renal cell cancer [218–220] Its dysregulation is associated with obesity and adiposity-related disorders including, binge‐eating disorders, anorexia and bulimia nervosa and diabetic Mellitus [26, 29, 30, 35, 36, 79, 81, 125]
AP-2γ/ *TFAP2C*	Kidney, neural crest cells, and human villous cytotrophoblast cells [42, 244]	As a negative regulator of the other AP-2 family members [42] Play a key role in trophectoderm development [244, 264] Regulates key genes responsible for eyes, face, and limb formation or neural tube development[115] Control the expression of other genes such as *FOXA1, WWOX, GREB1, CDH2, HPSE, IGSF11* and others [263]	Mutation causes Exencephaly, melanoma and pre-eclampsia [115] Act as an oncogenic factor in many cancers such as melanoma. neuroblastoma, breast, testicular and lung cancers [115, 266–269]
AP-2δ /TFAP2D	Brain, placenta, skeletal muscle, thymus, small intestine, retina, heart, and prostata [270, 271]	Important during embryogenesis and in retina development [271, 272] Regulation of the *HMOX1* gene[273]	Associated with aggressive prostate cancer[274]
AP-2ε /TFAP2E	Neural tissue of the midbrain and hindbrain [275], cells and vomeronasal sensory neurons of the olfactory bulb [276, 277], and cartilage [278]	Important for development of GABAergic interneurons in olfactory bulb [276, 277, 279] Regulation of integrin α10 transcription and the core promoter of type II collagen (COL2A1) Crucial for chondrogenesis and the development of cartilage during embryogenesis [278, 280, 281]	Associated with colorectal cancer [282] and neuroblastoma [263, 283] Implicated in hypertrophic cartilage and the development of osteoarthritis [278]

*FOXA1* Forkhead Box A1, *WWOX* WW Domain Containing Oxidoreductase, *GREB1* Growth Regulating Estrogen Receptor Binding 1, *CDH2* Cadherin-2, *HPSE* Heparanase, *IGSF11* Immunoglobulin Superfamily Member 11, *HMOX1* Heme Oxygenase 1, COL2A1 Core promoter of type II collagen

There are two DNA binding sites for AP-2: 2 cis-acting DNA sequences 5′-(G/C)CCCA(G/C)(G/C)(G/C)-3′ and the palindromic sequence 5′-GCCNNNGGC-3′ [27, 46, 47]. Except for AP-2δ, the transactivation domain of AP-2 proteins is characterized by a PY motif (XPPXY) and other highly conserved residues in the protein binding domain [48]. Despite structure similarities (76% homology) between AP-2α & AP-2β [49], their gene mutations give rise to different phenotypes (Table 1). Moreover, AP-2α-null mice exhibit severe craniofacial defects while AP-2β KO mice display massive apoptosis of renal cells and terminal renal failure [49–52]. This might indicate that they are involved in differential signalling pathways. Although the crystal structure of each AP-2 protein has not yet been established, a highly accurate prediction of their protein structures has been produced by AlphaFold [53] and is available at Uniprot (Q92481).

AP-2 transcription factors are prominent regulators of multiple genes involved in embryonic neural crest development, cell differentiation and haemostasis in a range of tissues but mainly in central and peripheral nervous and urogenital systems [42, 43, 55]. They mediate induction of the target genes in response to two signal-transduction mechanisms: phorbol-ester- and diacylglycerol-activated protein kinase C (PKC) and cAMP-dependent protein kinase A (PKA) [30, 51, 56, 57]. AP-2 transcription factors can act both as transcriptional activators or repressors of the target genes; therefore, we suggest that the name 'transcription factor activating protein 2 (AP-2)' used in the literature is actually a misnomer and should be considered as 'transcription factor trans-regulator protein 2 (TP-2)'.

### AP-2β transcription factor

AP-2β was first characterized in 1995 [41]. Two splice variants for AP-2βhave been identified, both can exert transcriptional regulatory activity but have a little different in tissue expressions: one short at 198 aa (22.1 kDa) (UniProt ID X6R4Y8), which is not well studied, and one long at 460 aa (50.5 kDa) (UniProt Q92481) that has been thoroughly investigated [58, 59]. AP-2β is highly expressed in the brain and peripheral neurons, eyes, smooth muscles of ductus arteriosus, skin, bone marrow, adipose and lymphoid tissues, proximal digestive tract, the adrenal medulla of the kidney and across the urogenital system [36, 58, 60]. In addition to its function in monoaminergic neurons, *TFAP2Β* is also involved in specification of glycinergic and GABAergic interneurons [61–63]. *TFAP2Β* mutations lead to the development of severe disorders such as craniosynostosis, Char Syndrome, dental anomalies, defects in patent ductus arteriosus, terminal renal failure and others [64–67] (Table 1).

## Regulation of AP-2β activity

**Fig. 2:**
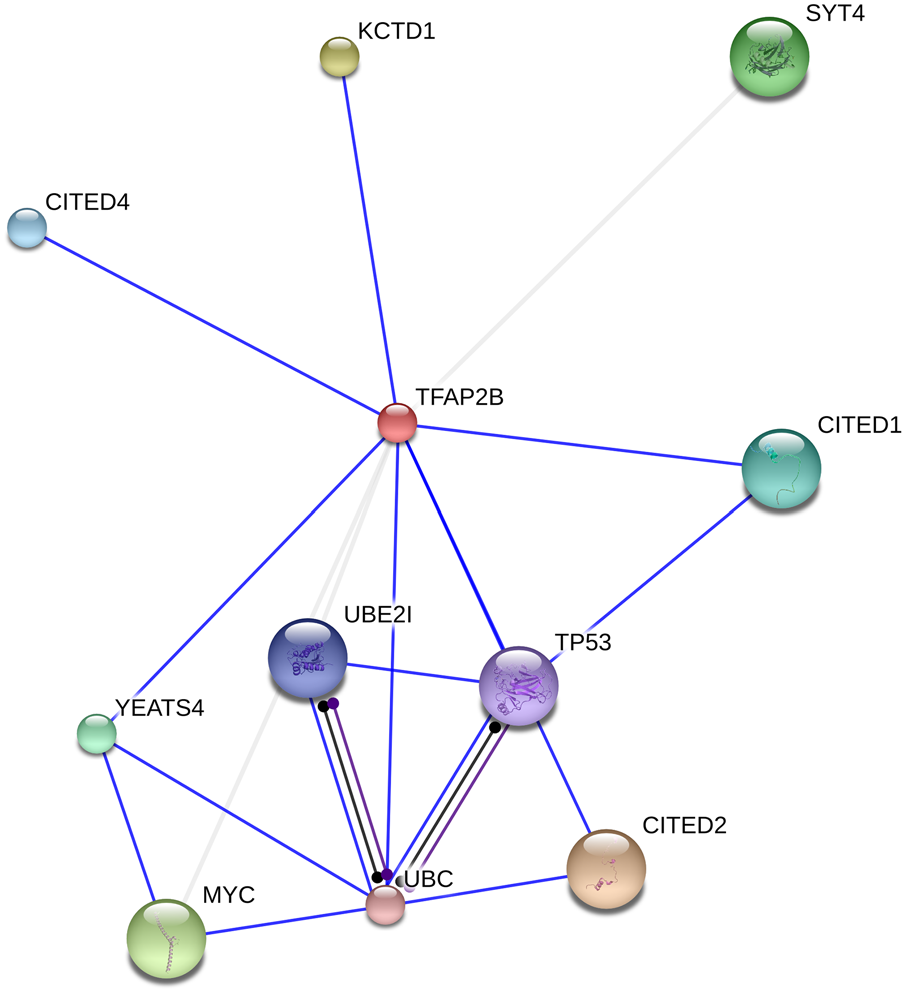
Protein interaction network of AP-2β factor. The image was taken from the STRING database [76] where the active interaction source is only experiments and the interaction score is of medium confidence (0.4). The blue line indicates binding, the purple catalysis, and the black reaction. YEATS4: YEATS domain-containing protein 4; KCTD1: potassium channel tetramerization domain 1; MYC: MYC proto-oncogene; TP53, cellular tumour antigen p53; UBC: ubiquitin carrier protein 9; UBE2I, ubiquitin-conjugating enzyme E2 I; SYT4: Synaptotagmin-4; CITED2 & CITED4: Cpb/p300-interacting transactivator 2 & 4

To better understand its associated MNDs, the mechanisms regulating AP-2β activity should be first articulated. Two key mechanisms have been shown to modulate the DNA-binding and/or transcriptional activities of AP-2β: protein–protein interactions through the protein-binding domain and post-translational modifications employing the DNA-binding domain (Figs. 1 and 2).

### Protein–protein interactions

AP-2β has a specific protein-binding domain, also known as a transactivation domain [44, 45, 67]. Binding to this domain modulates the DNA-binding ability of AP-2β at the adjacent DNA-binding domain [67]. A study by Ding et al. has shown, for example, that the potassium channel tetramerization domain 1 (KCTD1) binds to the N-terminal protein-binding domain of AP-2β and inhibits its transcriptional activity in human cell lines [68]. Another study by Zarelli et al. has reported that in zebrafish, potassium channel tetramerization domain 15 (KCTD15) inhibits the expression of AP-2 [69]. Interestingly, KCTD1 and KCTD15 have remarkably similar amino acid sequences [70], which may explain the similarity of their effects on AP-2β. Additional inhibitors of AP-2β transcriptional activity are the protein kinase D (PKD) [71], which, by phosphorylating AP-2β, inhibits its DNA binding activity, as well as the hypoxia-inducible factor-2alpha (HIF-2α), which negatively regulates the expression AP-2β [72].

The dynamics of protein–protein interaction can also lead to enhanced AP-2β activities. Cpb/p300-interacting transactivator 2 & 4 (CITED2 & CITED4), are coactivators and enhancers of AP-2β-induced transcriptional activation [73, 74]. Additionally, YEATS domain-containing protein 4 (YEATS4, also known as GAS41) augments both, the DNA-binding and the transcriptional activity of AP-2β [75]. For more information about other protein interactions with AP-2β, see Fig. 2.

### Post-translational modifications

Post-translational modification is another mechanism that can modulate the transcriptional or DNA-binding activity of AP-2β. For example, the sumoylation of AP-2β by ubiquitin carrier protein 9 (UBC9) has been shown to decrease its transcriptional activity [77]. KCTD15   mentioned earlier can also enhance the activity of AP-2β through sumoylation [78, 79]. Accordingly, KCTD15 can modulate the activity of AP-2β directly (blocking) and post-translationally (activating) (Table 2).

**Table 2:** The coactivators and suppressors of AP-2β

Suppressor	Coactivators/positive regulators
KCTD1 [68]	KCTD15 sumoylation [78, 79]
KCTD15 [69]	CITED2 [73]
UBC9 sumoylation [77]	CITED4 [74]
PKD phosphorylation [71]	YEATS4 [75]
HIF-2α [72]	

KCTD1: the potassium channel tetramerization domain 1; KCTD15: potassium channel tetramerization domain 15; PKD: protein kinase D; HIF-2α: inducible factor-2alpha; CITED2: Cpb/p300-interacting transactivator 2; CITED4: Cpb/p300-interacting transactivator 4: YEATS4; YEATS domain-containing protein 4; UBC: ubiquitin carrier protein 9; UBE2I: ubiquitin-conjugating enzyme E2 I

In summary, different modulators have been shown to regulate the activity of the AP-2β by two mechanisms: the protein–protein interaction through the transactivation domain and post-transcriptional modification through the DNA-binding domain. These two mechanisms could be utilized to pharmacologically modulate AP-2β activity, possibily by targeting these modulators.

## AP-2β signalling regulation of monoaminergic neurotransmitter systems

AP-2β is a vital transcription factor for the proper development and function of the monoaminergic neurotransmitter systems [24, 62, 80]. It plays an essential role in the maturation of chromaffin cells, sympathetic neuronal differentiation, and regulation of monoaminergic transmission, including adrenergic, noradrenergic and dopaminergic, and serotonergic transmission, during both development and adulthood [24, 46, 62, 80–82]. Several key genes regulating the monoaminergic neurotransmitters' biosynthesis, degradation and synaptic activity have a recognition site for AP-2β in their promoter regions [21–23, 27, 83, 84]. Since AP-2β enhances both monoaminergic transmissions, AP-2β levels correlate with monoamine neurotransmitter transmission in the brainstem [24, 25, 62]. This might explain why the central level of AP-2β has been changed after treatment with certain drugs that act on monoaminergic neurotransmitters, such as phenelzine (MAOI) and citalopram (SNRI) [24, 38, 85].

### AP-2β regulation of catecholaminergic transmission

**Fig. 3:**
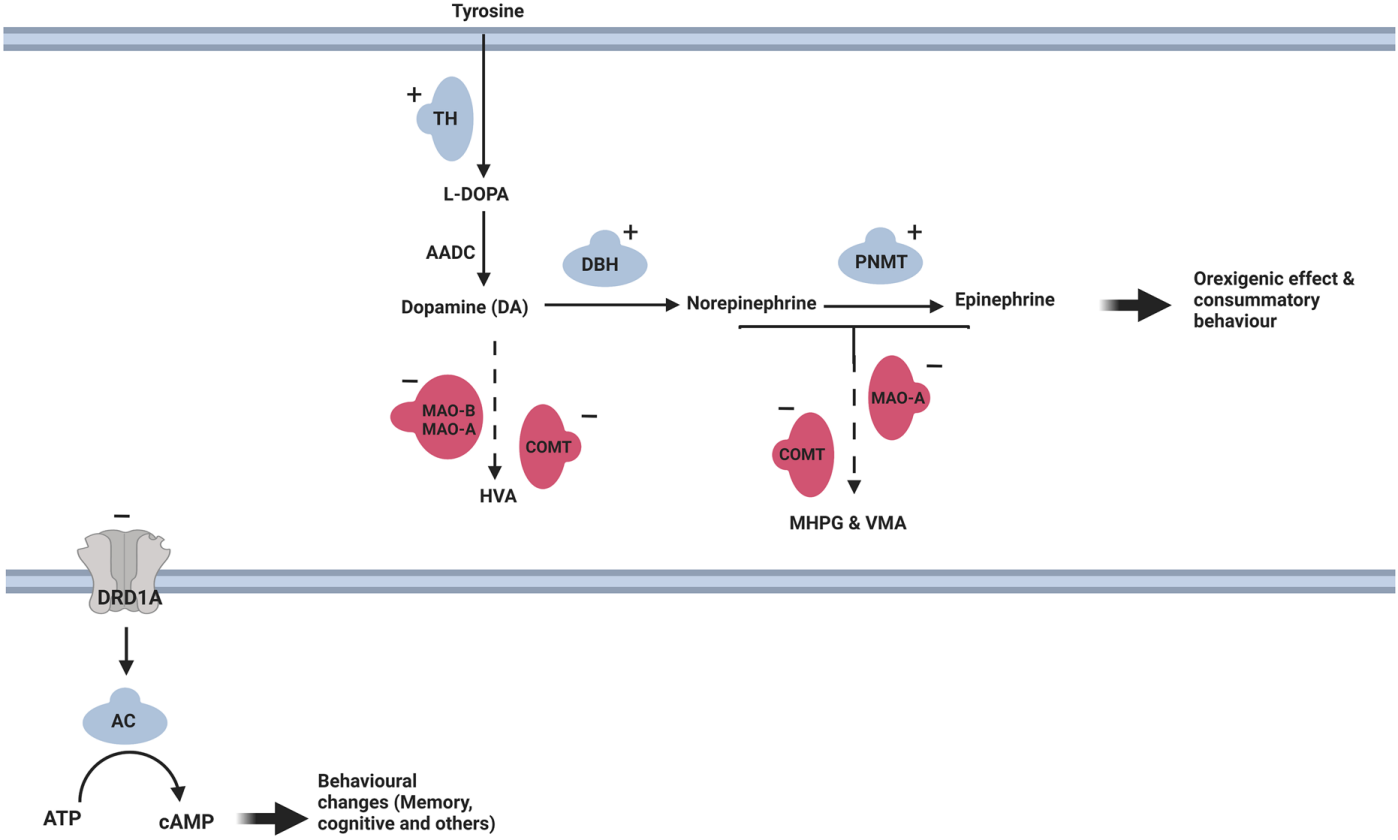
The signalling pathway of AP-2β on catecholaminergic transmission where '–' symbolizes repressing and '+ ' activating effect of AP-2β on the transcription of its target genes. AP-2β stimulate the expression of key genes involved in catecholamine synthesis such as *TH* [33, 82, 89]*, DBH* [33, 46, 82, 89, 90]*, PNMT* [46, 86–88] while it lowers genes coding catecholamine degrading enzymes such as *MAO* [28, 91–93] and *COMT* [21, 92]. AP-2β also repress the transcription of DRD_1A_ through binding to its D1AS1 [94]. AP-2β: transcription activating protein 2 beta; *TH*: tyrosine hydroxylase; *DBH*: dopamine-beta-hydroxylase; *PNMT*: phenylethanolamine *N*-methyltransferase; *MAO*: monoamine oxidase; *COMT*: catechol-*O*-methyltransferase; HVA: homovanillic acid; VMA: 3-methoxy-4-hydroxymandelic acid; MHPG: 3- methoxy-4-hydroxy-phenylglycol; D1AS1: dopamine receptor 1A silencer 1; *DRD1A*: dopamine receptor 1A; AC: adenylyl cyclase; and cAMP: cyclic adenosine monophosphate

AP-2β plays a crucial role in the regulation of catecholamine levels in the brain by altering the expression of several key genes in the catecholaminergic pathway (Fig. 3). AP-2β enhances adrenergic transmission, boosting norepinephrine and epinephrine availability [24, 46, 86–88]. It activates the transcription of the genes of catecholamine-synthesizing enzymes, such as tyrosine hydroxylase (*TH)* [33, 82, 89], dopamine-beta-hydroxylase (*DBH)* [33, 46, 82, 89, 90] and phenylethanolamine *N*-methyltransferase (*PNMT*) [46, 86–88] Furthermore, it lowers the genes of catecholamine-degrading enzymes, such as monoamine oxidase-A & B (*MAO-A)*, (*MAO-B)* [28, 91–93] and *COMT* [21, 92].

Furthermore, AP-2β represses the Dopamine receptor D_1A_ (DRD_1A_) receptor through binding to the D_1A_ silencer 1 (D1AS1) [94]. Blocking this receptor leads to decreased adenylyl cyclase (AC) activity and lowered cyclic adenosine monophosphate (cAMP), resulting in dopamine-related behavioural changes such as reward, cognition and learning [94–96] (Fig. 3). DRD1_A_ plays a significant role in the modulation of memory and cognition [97–99]; hence, it would be interesting to explore the association of *TFAP2B* polymorphisms with cognitive functions.

The effect of AP-2β on dopamine turnover is under debate. Damberg et al. have reported that AP-2β is positively correlated with the indices of dopamine metabolites, namely homovanillic acid (HAV) and 3-methoxy-4-hydroxy-phenylgly (MGPG) in rat forebrain [24] but not in CSF of humans [25]. Moreover, DBH enzyme is responsible for converting dopamine into norepinephrine in catecholaminergic neurons [100]; thus, given its transcriptional activation effect on *DBH *[33, 46, 82, 89, 90], AP-2β might enhance the turnover of dopamine by boosting its conversion into norepinephrine. On the other hand, Schabram et al. have suggested that AP-2β enhances dopamine availability and decreases its turnover through its repressing effect on *MAO-A* and *COMT* [92]. In total, it is difficult to conclude the influence of AP-2β on dopamine levels and hence, more investigations in this area are recommended, especially in the AP-2β-associated MNDs where dopamine is a key player, e.g. obesity and anxiety.

### AP-2β regulation of serotonergic transmission

**Fig. 4:**
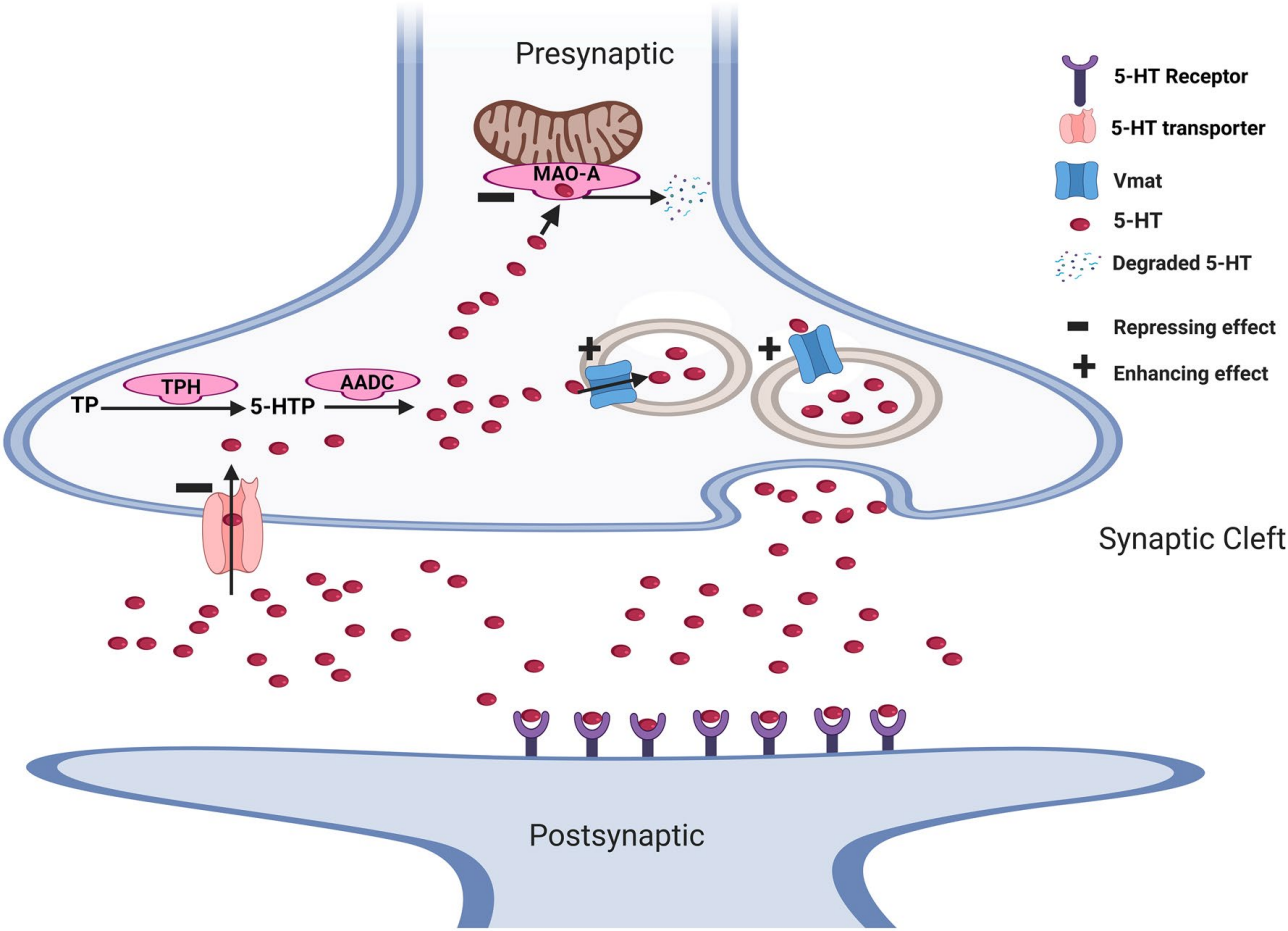
The effect of AP-2β on serotonergic transmission in the brain. AP-2β enhances the 5-HT levels in the synaptic cleft by increasing the transcription *Vmat* [81] and repressing *5-HTT* [19, 20, 101] and *MAO-A* [28, 91–93]. AP-2β has binding sites on *TPH* & *AADC* genes and *5-HT* receptor [62, 102–107], but its effect on them is unknown. 5-HT: serotonin; *Vmat: Drosophila* vesicular monoamine transporter; *5-HTT:* 5-HT transporters; *MAO-A*: monoamine oxidase-A; *TPH*: tryptophan hydroxylase; *AADC*: aromatic L‐Amino acid decarboxylase; *5-HT* receptor: serotonin receptor

AP-2β controls multiple key genes in the serotonergic (5-HT) pathway (Fig. 4) and has been shown to enhance serotonergic turnover and transmission in the brain [24], perhaps by increasing serotonin availability in the synaptic cleft. Two key genes in the serotonergic system have been shown to be negatively regulated by AP-2β: *MAO-A* [28, 91–93] and *5-HTT* [19, 20, 101]. 5-HTT transports 5-HT into presynaptic where it is degraded by MAO. Thus, inhibiting 5-HTT and MAO genes by AP-2β may result in increased serotonin levels in the synaptic cleft and serotonergic transmission [24, 25]. In addition, AP-2β has a binding site on tryptophan hydroxylase (*TPH*), aromatic l‐Amino acid decarboxylase (*AADC*) as well as rat *5-Htr2* and human *5HT3R* [62, 102–107]; yet, its regulatory effect on these genes is still unknown. Given the positive correlation between AP-2β and serotonergic activity in the brainstem [24, 25], it is reasonable to suggest that AP-2β may enhance the expression of *TPH* and *AADC* as well, although studies are needed to confirm this suggestion.

The vesicular monoamine transporter 2 gene (*VMAT2*), a key regulator of the monoamine neurotransmitters' availability in the synaptic cleft, has a putative AP-2β binding site in its promotor region [23]. However, the regulatory effect of AP-2β on this gene has not been fully explored. In an attempt to explore this, using *Drosophila melanogaster*, our group has shown that the *Drosophila* analogue of *TFAP2Β*, *TfAP-2*, positively regulates the expression levels of *Drosophila VMAT2*, *Vmat*, and activates octopaminergic neurons (analogous to human noradrenergic neurons) [81]. Considering that *TFAP2Β* is conserved in *Drosophila* (*TfAP-2*) and there is a high homology between *Drosophila Vmat* and human *VMAT2* (weighted score 14/15) [108, 109], AP-2β might also activate *VMAT2* in humans. Consistent with this suggestion is the observations that, similar to AP-2β [24, 25], activation of *VMAT2* enhances monoaminergic transmission in the synaptic cleft, not to mention that *VMAT2* dysregulation results in disorders that are also associated with *TFAP2Β*, namely depression [110], AD [111], alcoholism [112] and obesity [113, 114]. Altogether, more research on AP-2β effects on human *VMAT2*, for instance in human neuronal cell culture, are warranted.

Taken together, AP-2β can modulate both arms of monoaminergic neurotransmitter systems, by controlling the key genes in catecholaminergic and serotonergic transmission, indicating its role as a key regulator of monoaminergic neurotransmitter systems and its importance for better understanding and management of MNDS.

## TFAP2Β polymorphisms

The *TFAP2Β* gene is located on chromosome 6p12-p21, and it has eleven axons with a size of 29,910 bases [115, 116]. *TFAP2B* has many polymorphic regions that affect mostly its transcriptional activity rather than its protein structure [29, 34, 117]. This in turn influences the expression of its target genes in the monoaminergic neurotransmitter systems. Consequently, *TFAP2Β* functional polymorphisms have been associated with different MNDs. To date, three functional polymorphisms of *TFAP2Β* are known to be associated with MNDs. One is located in intron 1, in which polymorphic regions (SNP at intron 1 + 774T/G and a nearby variable number tandem repeat, VNTR, allele), have been shown to postively regulate *TFAP2B* transcriptional activity, enhancing  its expression. Subjects who carry the disease-related alleles (S-allele: T-nucleotide for SNP, nine repeats for VNTR) show higher expression of *TFAP2Β*, which is associated with a higher risk for T2D and a lower risk for depression [34, 36, 102, 118].

The second polymorphism is located in intron 2 in a polymorphic region [CAAA] close to the 3`-splice site of exon 2 between nucleotides 12593 and 12612 [27, 51]. Repeats of this sequence in variable regions suggest its important role in functional polymorphisms [25, 27, 60]. Interestingly, the 5-repeat alleles of indel intron 2 of *TFAP2B* have been shown to increase the expression of *TFAP2Β* [19]. The intron 2 polymorphism is associated with adiposity, neonatal temperament and anxiety-related personality traits [19, 26, 27, 29]. Strikingly, there is significant linkage disequilibrium between the polymorphisms of intron 1 and intron 2 [27, 29, 119], indicating that their association is not by chance. This suggests that the associations observed with intron 2 polymorphisms are most likely a result of the functional polymorphism in intron 1 [28].

The third *TFAP2Β* polymorphism, rs987237, is located in intron 3 and has three different genotypes, AA, AG and GG that are associated with obesity, BMI, waist circumference and differential effects on weight loss [120–123]. Stocks et al. have suggested that the *TFAP2Β* rs987237 variant may be linked to enhanced activity of this transcription factor since it is also in complete linkage disequilibrium with intron 1 [120].

Overall, three TFAP2Β polymorphisms have been shown to mostly enhance its transcription activity. This in turn affects the expression of the key genes in the monoaminergic neurotransmitter systems that have putative binding sites for AP-2β.

## Monoamine neurotransmitter disorders associated with *TFAP2Β* dysregulation and possible underlying mechanisms

### Obesity and type 2 diabetes (T2D)

Studies have shown that *TFAP2Β* polymorphisms of intron 1,2 & 3 that lead to higher expression of *TFAP2Β*, are associated with reward fixation, insulin resistance, T2D and adiposity, lipid droplet biogenesis as well as eating disorders [29, 34, 36, 124–128]. GWAS studies have indicated an association of *TFAP2Β* rs987237 within intron 3 with obesity, BMI and waist circumference in women [120, 129, 130]. There is, however, a debate whether rs987237 is associated with improved weight loss, depending on the genotype (AA, GG or AG) [120–122] and the type of diet (high/low-fat diet or high/low protein diet) [120, 131].

The mechanisms underlying AP-2β-induced insulin resistance and adiposity are rather unclear. Several studies have attributed the role of AP-2β in insulin resistance and adiposity to its regulatory effect on genes that encode adipocytokines, which have AP-2β binding sites in their promoters. For instance, *TFAP2Β* overexpression has been shown to inhibit the expression of adiponectin and leptin but also enhance the expression of interleukin 6 (IL-6), monocyte chemoattractant protein-1 (MCP) and tumour necrosis factor-alpha (TNF-alpha) [34, 124, 132–135]. However, Tsukada et al. have demonstrated that overexpressed *TFAP2Β* has no effect on most adipocytokines like adiponectin, leptin and IL-6 [34]. Additionally, a study by Nordquist et al*.* have shown no association of adiponectin levels to insulin resistance or to the *TFAP2Β* polymorphism [29]. Furthermore, AP-2β has been reported to enhance glucose uptake (enhancing the translocation of the glucose transport 4, GLUT4) but also reduce insulin sensitivity through both, repressing the expression of insulin receptor substrate 1 (IRS-I) and lipid accumulation [29, 31, 36, 133, 134]. The enhanced glucose uptake effect, however, is not in line with the inhibitory effect of AP-2β on adiponectin and leptin mentioned above since higher adiponectin and leptin have been shown to enhance glucose uptake [136, 137]. Given such discrepancies, it is reasonable to suggest another pathway by which AP-2β contributes to obesity and T2D, possibly through monoaminergic neurotransmitter systems.

Many research studies have revealed that polymorphisms or overexpression of *TFAP2Β* contribute to eating disorder-related behaviour through catecholamine-induced orexigenic signals [78, 79]. In terms of glucose uptake and insulin resistance, AP-2β augments norepinephrine availability [24, 25], and elevated norepinephrine has been reported to increase glucose uptake and transport in adipose tissue [138, 139]. Moreover, AP-2β increases both norepinephrine and epinephrine levels [24, 25], both of which can contribute to insulin resistance and obesity [140–145]. Furthermore, AP-2β upregulates *PNMT* [46, 86–88], and upregulated *PNMT* has been associated with elevated epinephrine and reduced circulating leptin levels [146], both of which can give rise to obesity [141, 142, 146].

The association of *TFAP2Β* with obesity-related disorders might also be linked to its repression effect on *5-HTT*. First, 5-HTT re-uptakes 5-HT from the synaptic cleft into the presynaptic neurons. As we mentioned previously, AP-2β represses the expression of *5-HTT* [19, 20, 101] and* 5-HTT* gene promotor methylation is associated with reward and eating behaviour as well as human obesity [147] and suppression of *5-HTT*, by hypermethylation, in humans is associated with a high prevalence of obesity [148]*.*

The genetic interaction between *TFAP2Β* and *KCTD15*, an obesity-linked gene, is also likely to positively contribute to AP-2β-induced obesity and metabolic dysfunction. In this regard, our group has recently shown that in *Drosophila*, the homologue of *KCTD15*, *Twz*, genetically interacts with *Drosophila TfAP-2* to regulate octopaminergic (human noradrenergic) signalling. We have further demonstrated that mouse *TFAP2β* and *Kctd15* directly interact with Ube2i, a mouse sumoylation enzyme, to induce consummatory behaviour [79]. Strikingly, consistent with these findings is a recent study by Gamero-Villarroel et al*.* that has identified the genetic interaction between *KCTD15* and *TFAP2Β* in individuals with eating disorders [78]. The same study has suggested that after sumoylation of *TFAP2Β* by *KCTD15*, a post-translational effect is initiated by E1‐3 enzymes and ATP, resulting in elevated catecholamine levels and induction of feeding behaviour [78].

### Alcoholism

That dysregulation of monoamine neurotransmitters contributes to the development of alcoholism is well known. Reports have indicated that *TFAP2Β* polymorphism in the intron 1, which can enhance AP-2β transcription, is associated with severe alcoholism in women [28]. This association has been linked to the effect of AP-2β on several targets in the monoaminergic system. AP-2β lowers the levels of MAO and lowered levels of MAO are linked to alcoholism [28, 149–151]. AP-2β activation of dopamine-beta-hydroxylase (*DBH)* [82] may also contribute to alcoholism, since in alcohol-dependent persons, *DBH* is hypomethylated and its enzyme is more active, resulting in a reduction of dopamine levels [152, 153]. However, inhibition of DRD1 or *DRD1* KO mice has been shown to reduce alcohol-seeking behaviour [154–157], suggesting that AP-2β-induced alcohol intake might be independent of its repressing effect on DRD1 [94].

Moreover, AP-2β-enhanced serotonergic transmission may also contribute to alcohol abuse, perhaps through inhibition of *5-HTT* [19, 20, 101] and lowering *MAO* [28, 91–93]*.* In agreement with this notion, increased serotonin levels or *5-HTT* KO mice have been found to trigger alcoholism [158–160], and *MAO-A* methylation is associated with alcoholism in women [161]. Furthermore, alcoholics have higher levels of both, the transcript and protein of tryptophan hydroxylase, *TPH* [162], the rate-liming enzyme in serotonin synthesis, which has a binding site for AP-2β in its promotor [107]. Increased levels of TPH have been reported to enhance serotonin levels [163].

### Anxiety and depression

*TFAP2Β* intron 2 polymorphisms are associated with low anxiety [26, 27, 93], and its intron 1 polymorphism, which enhances its expression, protects against the risk of depression in patients with attention deficit hyperactivity disorder [102]. However, when psychosocial adversity is considered, individuals with *TFAP2Β* intron 2 polymorphisms who are homozygous for the short *TFAP2Β* allele exhibited higher depression scores [117].

This association might be ascribed to the AP-2β regulatory effect on the monoaminergic neurotransmitters in the brain. Mechanistically, AP-2β inhibits 5-HTT expression [19, 20, 101] and inhibition of 5-HTT, by SSRIs for instance, leads to increased serotonin, which alleviates anxiety and depression [164]. Moreover, AP-2β reduces MAO levels [28, 91–93], which may also contribute to reduced anxiety and depression. In agreement with this possibility, deficiency of MAO-A or MAO-B leads to reduced anxiety-like behaviour in mice, as well as inhibition of MAO-A reduces depression in mice, and likewise, drugs that inhibit MAO-B reduce depression as well [165, 166]. Interestingly, MAO inhibitors enhance the levels of noradrenaline and serotonin levels in the brain to alleviate anxiety [167] and depression [168]. In consonance with these effects, AP-2β enhances noradrenaline and serotonin transmission [24], both of which have been reported to exert anxiolytic and antidepressant effects [9, 169–171].

Since certain antidepressant/anxiolytic drugs, e.g. citalopram (SSRI) and imipramine (TCA), have been reported to modulate the levels of AP-2β in the brainstem of the rat [24, 38, 84], probably due to its transcriptional regulation of the key targets in monoaminergic neurotransmitter systems, future clinical  anxiolytic/antidepressant drug development should monitor the level of AP-2β for better management and/or prevention of depression and anxiety.

### Antisocial behaviour

Antisocial behaviour in children and adolescents refers to a heterogeneous set of actions outside the norms of society, including aggression, impulsive behaviour and criminal acts, which are linked to monoaminergic neurotransmitter systems [172–175]. *TFAP2Β* has been associated with aggression in fruit flies and humans, as well as with general antisocial behaviour in humans [27, 28, 81, 176]. However, the *TFAP2Β* signalling mechanisms that underlie such association have not been fully elucidated. The repressing effect of AP-2β on monoamine-degrading enzymes, such as *COMT* and *MAO* might explain such an association since several reports have revealed that lowered expression of *MAO-A*, or *MAO-A* KO mice, as well as lower activity of COMT or COMT-deficiency in mice are associated with higher aggression [177–182]. Furthermore, activation of *DBH* expression by AP-2β may also play a part in provoking high aggression. Activation of *DBH* enhances the conversion of dopamine to adrenaline and it has been reported that higher adrenergic signalling provokes aggression [183], and *DBH* KO mice have lower levels of aggression [184], all linking AP-2β-mediated higher aggression to enhanced adrenergic signalling.

Nevertheless, the AP-2β association with higher aggression might be independent of its enhancement effect on serotonergic signalling, specifically, its repressing effect on *5-HTT*. This is because several preclinical and clinical studies have indicated that high levels of serotonin and lower expression or blocking of 5-HTT or knocking out *5-HTT* in mice lead to reduced aggression outbursts and violent behaviour [185–188], an effect opposite to that of AP-2β*.* It would be interesting to investigate the association of *TFAP2Β* polymorphisms and their interaction with key monoamine neurotransmitter genes to aggression and antisocial behaviour.

### Alzheimer's disease (AD)

While genetic variants of *TFAP2Β* have been suggested to play a role in resilience to AD [189], increased AP-2β has been shown to bestow a neuroprotective effect in AD due to the AP-2β-enhancing effect on the expression of apolipoprotein E (apoE), an important protective protein in AD pathogenesis [32, 190]. The effects of AP-2β on catecholamines might also contribute to its protective effect in AD. Firstly, lowered catecholamine levels contribute to the development and pathogenesis of AD [191–193] and, by the same token, AP-2β has been shown to increase catecholamine signalling in the brain [24, 46, 86–88]. AD is also associated with lower levels of *DBH* and *PNMT* [194–198], which both are activated by AP-2β [46, 86–88]. In addition, COMT and MAO-B levels are higher in AD [192, 199–201], and both are lowered by AP-2β [21, 92, 93]. Supporting this notion, COMT and MAO inhibitors have been repurposed for the treatment of AD [202–204]. Secondly, AP-2β enhances serotonin activity in the brain which might add further protective effects in AD. Consistent with this extrapolation is the observation that reduced serotonin levels in the brain can enhance the risk for AD [205], providing the rationale for SSRI use to delay the onset of AD [206]. Taken together, it might be suggested that, through its enhancing catecholaminergic and serotonergic activities, AP-2β may exert a protective role in AD. Thus, elucidating the association of *TFAP2Β* polymorphisms and their interactions with *COMT*, *MAO*, to AD could unveil a potential biomarker for early diagnosis and management of AD.

### Neuroblastoma

Transcription factors serve as essential regulators of cell development, proliferation and differentiation; consequently, dysregulation of them brings about oncogenic transformation and cancers [207, 208]. Neuroblastoma is an embryonal pediatric malignant tumour originating from the sympathetic nervous system and characterized by extremely low noradrenergic neuronal differentiation [33]. However, the molecular mechanisms underlying lowered neuronal differentiation in neuroblastoma are still under investigation.

Recent studies have revealed an important role of AP-2β in the pathogenesis and progression of neuroblastoma [33, 37, 209, 210]. Thorell et al*.* have identified *TFAP2Β* as a potential tumour suppressor gene in neuroblastoma [210]. Ikram et al*.* have also indicated that low AP-2β expression results in lower noradrenergic neuronal differentiation and is thereby significantly associated with poor prognostic markers and unfavourable patient outcomes [33]. In contrast, induction of AP-2β expression has been found to impair tumour cell proliferation and slow tumour progression by enhancing both differentiation of noradrenergic neurons as well as noradrenergic signalling through increased expression of *TH* and *DBH* [33]. In addition to its effects on sympathetic neurons, AP-2β has been demonstrated to enhance retinoic acid (RA) responsiveness, which potentiates neuronal differentiation and therefore is used in neuroblastoma therapy [33].

Altogether, AP-2β is associated with certain MNDS, although the underlying mechanisms are not fully elucidated. As we mentioned above, the underlying mechanisms for this association might be linked to AP-2β regulatory effects on key genes of monoaminergic neurotransmitters. However, further preclinical studies investigating the molecular mechanisms underlying AP-2β associated MNDs, e.g. manipulating the expression of *TFAP2B* in neuronal cell culture or rats’ models of MNDs and examining the levels of the key enzymes of monoaminergic neurotransmission, are warranted.

## AP-2β as a biomarker and a potential therapeutic target

### AP-2β as a biomarker

Transcription factors are currently widely used as diagnostic biomarkers for the early detection of several diseases [211–213]. A wide range of brain-related disorders and cancers are accompanied by alterations in the levels and activity of *TFAP2Β*/AP-2β, which underscores its diagnostic importance for such diseases. *TFAP2Β* overexpression, for instance, has been implicated not only as a protective or favourable prognostic factor in several cancers like breast, renal cell, cervical and endometrial cancers but also as a poor prognostic factor in thyroid cancer and lung adenocarcinoma [214–225] (See Table 1).

Along the same line, AP-2β might also constitute a diagnostic biomarker for other MNDs. For example, while *TFAP2Β*/AP-2β overexpression has already been suggested as a favourable prognostic marker in neuroblastoma[33, 210], it might further be considered as a biomarker for other MNDs where *TFAP2Β*/AP-2β overexpression is implicated, such as alcoholism, obesity, T2D and aggression. Nevertheless, clinical studies are needed to address its specificity and characterize its validity as a biomarker for the early diagnosis of these disorders.

### AP-2β as a potential therapeutic target

In the last decade, targeting transcription factors with selective therapeutic agents has gained growing interest because transcription factors act as on/off switches of gene expression, a process that leads to neurological disorders and cancers when disrupted. In this context, clinical reports have highlighted the potential of AP-2β as a therapeutic target for specific cancers such as lung adenocarcinoma [216], renal cell tumorigenesis [220], and breast [221] and endometrial cancers [217].

*TFAP2Β*/AP-2β might also be considered an interesting therapeutic target for the management of specific MNDs, where its synergistic effects on multiple targets may be beneficial for the treatment or prevention of complex diseases. This concerns specifically the neurometabolic disorders, such as obesity and type 2 diabetes, that are characterised by widespread systemic alterations through diverse factors including, behavioural, neural, hormonal, adipose and intestinal along with the involvement of different mediators such as monoamine neurotransmitters, insulin and adipocytokines*.* In this regard, researchers have emphasized the importance of AP-2β as a promising drug target for the prevention and treatment of obesity and T2D [34–36]. AP-2β can target multiple features of obesity and T2D centrally and peripherally by modulating key-obesity linked genes such as *IRS-1*, *GLUT4*, adipocytokines related genes, as well as catecholaminergic and serotonergic genes involved in reward, consummatory behaviour and insulin resistance (Table 3). Some of these genes are, intriguingly, recognized as therapeutic targets of several drugs used for obesity-linked diseases.

**Table 3:** Summary of the mechanisms underlying AP-2β effects, and potential therapeutic interventions for specific monoamine neurotransmitter disorders (MNDs)

Monoamine Disorder	AP-2β effect	AP-2β target genes	Affected modulators	Possible pharmacological intervention	Possible therapeutics
Obesity and Type 2 diabetes	Induction	*TH*(+) [33, 82, 89] *DBH* (+) [33, 46, 82, 89, 90] *PNMT* (+) [46, 86–88] *5-HTT* (−) [19, 20, 101] *VMAT2* (?) [23] *DRD1*_*A*_ (-) [94] *MAO* (-) [28, 91–93] *COMT* (−) [21, 92] Adipokines-related genes (±) [34, 123, 131, 133–135] *IRS-1*(−) [134]	Norepinephrine (+) Epinephrine (+) Dopamine Serotonin (+) Adipocytokines (±) Insulin (−)	Inhibition of AP-2β	Peptide inhibitors
Neuroblastoma	Suppression	*TH* (+) [33, 82, 89] *DBH* (+) [33, 46, 82, 89, 90] *PNMT* (+) [46, 86–88]	Norepinephrine (+) Epinephrine (+) Dopamine	Activation of AP-2β	AP-2β analogue or activators Tetracycline [33]

*TH* tyrosine hydroxylase, *PNMT*: phenylethanolamine *N*-methyltransferase, *MAO* monoamine oxidase, *5-HTT* serotonin transporter, DRD1_A_: dopamine receptor D1A, *VMAT2*: Vesicular monoamine transporter 2, *DBH* dopamine-beta-hydroxylase, *IRS-1*: Insulin receptor substrate 1; *COMT* catechol-*O*-methyltransferase

A second possible application of AP-2β as a therapeutic target is in the treatment of neuroblastoma [37], where the benefits of treatment success could outweigh the possible side effects. As mentioned previously, lower expression of *TFAP2Β* is associated with a poor prognosis of neuroblastoma since patients whose tumour cells have lowered *TFAP2Β* showed poor treatment outcomes [33]. In contrast, elevated *TFAP2Β* has been reported to improve patient outcomes [33] by enhancing noradrenergic neuronal differentiation through different target genes in monoaminergic transmission [33, 210] and subsequently repress the progression of neuroblastoma. Most importantly, induction of *TFAP2Β* expression by tetracycline has already been successfully employed to impair tumour cell proliferation and slow neuroblastoma progression [33]. Collectively, boosting AP-2β levels might represent a potential therapeutic approach, perhaps in combination with other anti-neuroblastoma therapies, to treat neuroblastoma, possibly through a localized drug delivery, which could minimize peripheral undesired effects. This is supported by the fact that Trichostatin A, a potential drug used for neuroblastoma, has been shown to augment monoamine pools by inhibiting *COMT* and *MAO-A* genes [226], a mechanism similar to that of AP-2β.

Like other drug targets at the transcription level, off-target effects could pose a substantial challenge. AP-2β has been reported to modulate multiple key genes within and outside the monoaminergic neurotransmitter systems, suggesting off-target effects. Consequently, such ubiquitous off-target effects might limit its potential as a therapeutic target due to the lack of specificity and the risk of adverse effects.

### Targeting AP-2β (druggability), challenges and future insights

Until recently, transcription factors had been considered very challenging targets (undruggable) since transcription factors lack obvious druggable pockets and the transcription process is carried out in the nucleus; therefore, the therapeutic agent should have the appropriate physicochemical properties to cross many biological barriers and reach the nucleus with sufficient concentration. Moreover, many crucial components involved in the transcription process do not have the enzymatic activity adequate for pharmaco-chemical interventions [227, 228].

**Fig. 5:**
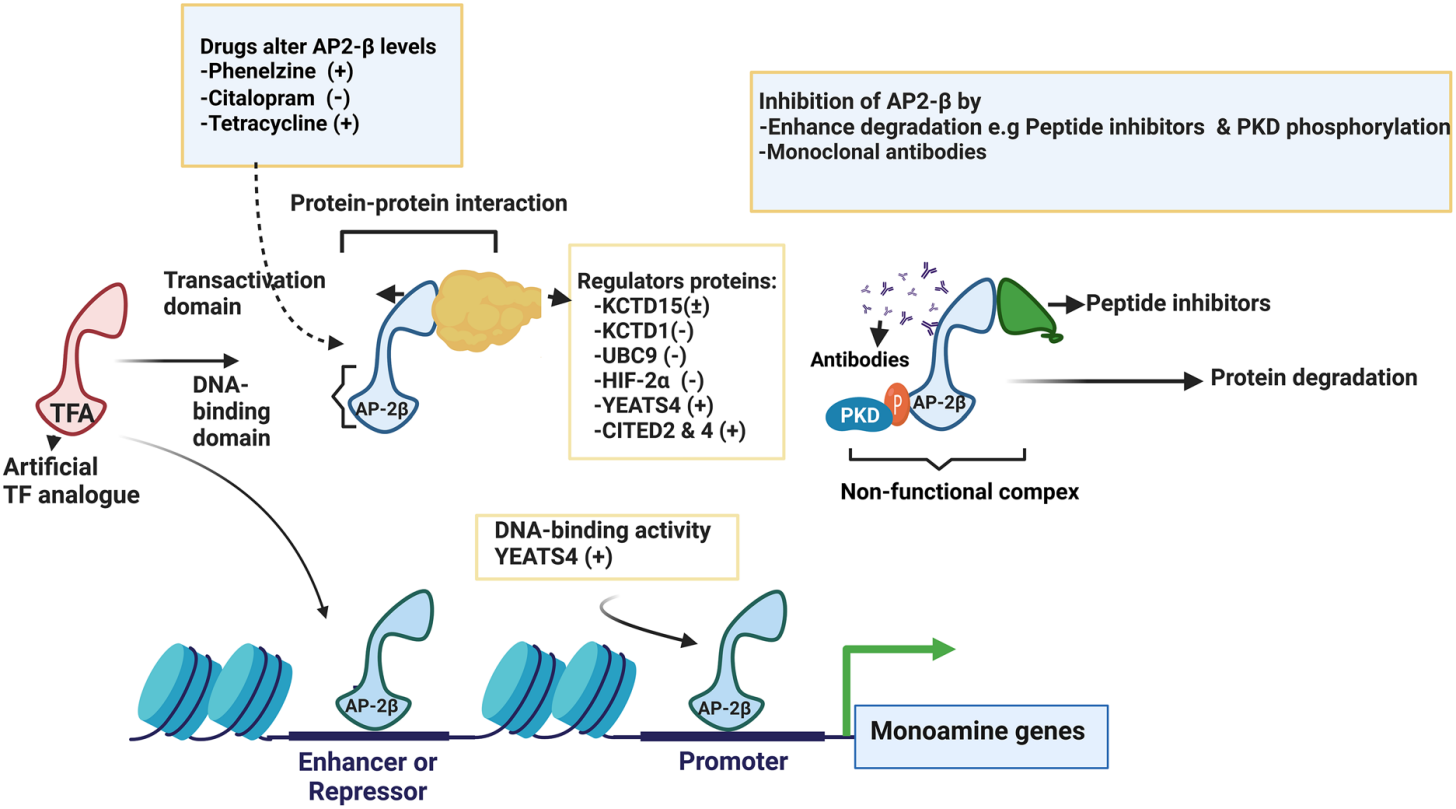
Possible sites and targets that can be potentially exploited to modulate AP-2β activities and/or levels. One proposal for modulating AP-2β is through protein–protein interactions whereby co-activators/suppressors bind to the transactivation domain and modify AP-2β transcription activity and DNA-binding activities, as indicated within the yellow square. Inducing degradation of AP-2β could be achieved through designing peptide inhibitors binding selectively to its transactivation domain to form a non-functional complex or by enhancing PKD phosphorylation of AP-2β [71] or by developing specific monoclonal antibodies that can bind and inactivate AP-2β. By contrast, enhancing AP-2β activity could be feasible by designing artificial transcription factor analogues (TFA) that can act as AP-2β agonists. Some monoaminergic drugs, such as phenelzine and citalopram, also have been shown to alter the brain levels of AP-2β [38, 39]] while tetracycline induces its gene expression[33]. KCTD1 & KCTD15: potassium channel tetramerization domain 1 & 15; UBC9: ubiquitin carrier protein 9; HIF-2α: hypoxia-inducible factor-2alpha; YEATS4: YEATS domain-containing protein 4; CITED2 & 4: Cpb/p300-interacting transactivator 2 & 4; PKD: the protein kinase D; DAG: diacylglycerol. The Figure was created with BioRender.com

So far, manipulation of transcription factor activity at protein–protein interaction levels has been successfully implemented and a few drugs have been approved for various disorders [227, 229]. As mentioned previously, several proteins have been reported to modulate the levels and activity of AP-2β (see Table 2). Such modulator proteins can be targeted to regulate *TFAP2Β*/AP-2β at different levels: at transcription, post-translation or the DNA-binding site (Fig. 5). Yet, potency and selectivity may pose a substantial issue because targeting these proteins can elicit a chain of inadvertent adverse effects since they are also involved in diverse biological processes and regulate other AP-2 transcription factors [42].

Despite these challenges, in the last decade, advances in pharmacological interventions have facilitated the druggability of transcription factors by specifically modulating their DNA-binding and transcription regulation activities at specific pockets. Interestingly, such pharmacological strategies have been successfully applied preclinically [228, 230–234] and clinically [235, 236], and at least 10% of drugs obtained FDA approval [208, 228, 237, 238]. Moreover, a recent article in Nature Reviews Drug Discovery by Henley et al*.* has discussed the renewed interest in the advent of pharmacological interventions for targeting transcription factors [208]. Currently, about ten drugs acting on transcription factors are in clinical trials [208], indicating a driving trend toward targeting transcription factors.

For example, inducing protein degradation of the transcription factor can be driven by exploiting its transactivation domain. Binding to this part with a specific peptide can form a non-functional complex for degradation. Such technology has facilitated abolishing of transcription factor activity by designing peptide inhibitors, also known as peptide therapeutics [233] that binds to the transactivation domain of the transcription factor and thereby hinder its interactions with other proteins and ultimately induce its degradation (Fig. 5). An interesting example in this context is YK-4-279, which peptide inhibitor that binds to the oncogenic transcription factor EWS-FLI1 to inhibit its activities. YK-4-279 is used to treat Ewing sarcomas [231, 239].

In parallel, providing the basic knowledge of its domain structure and binding sites, direct inhibition of AP-2β, by peptide inhibitors capable of selectively binding to its transactivation domain and inducing its degradation could be attractively applicable. Such specific peptide inhibitors could interfere with the dynamic of AP-2β protein–protein interactions and subsequently block its transcriptional activity on the target genes. This represents an interesting therapeutic approach for the management of obesity and related neurometabolic disorders where higher AP-2β levels are implicated.

On the other hand, mimicking transcription factor activity has been made feasible, especially in cancer and neurometabolic disorders. Many artificial transcription factors, also called transcription factor analogues (TFAs), have been successfully developed for several cancers and loss-of-function mutations to restore the overall functions of the transcription factors [231]. Dimethyl fumarate (DMF), for example, is an NRF2 activator, which is FDA-approved for multiple sclerosis (MS) [237]. In a similar vein, induction of AP-2β-related transcriptional effects on its target genes could be achieved by designing an AP-2β analogue with sufficient selectivity and potency (Fig. 5). Such AP-2β agonists could mediate effects similar to that of AP-2β and thereby be used in disorders where higher AP-2β is therapeutically advantageous such as in neuroblastoma. Nevertheless, full characterization of the protein crystal structure of AP-2β is a prerequisite for developing such therapeutic agents.

## Concluding remarks

AP-2β is a central regulator of monoamine neurotransmitters and its dysregulation is associated with MNDs. The underlying mechanisms for its associated MNDs could be linked to its regulatory role in monoaminergic transmission, yet more studies are recommended. For example, knockout/overexpression of *TFAP2B* in rat models of MNDs and examining the expression of the key monoaminergic genes can help clarify the molecular pathogenesis of the associated MNDs. Moreover, the molecular functions of AP-2β within the monoaminergic systems underline its importance as a promising biomarker for the early diagnosis of relevant MNDs. Given its effect on multiple targets within and outside monoaminergic systems, AP-2β could be considered a dirty drug target due to the odds of having adverse effects. Nevertheless, several dirty drugs that aim for multiple targets at once are clinically effective in the treatment of complex diseases [240, 241], which might also underscore the therapeutic potential of the AP-2β for complex MNDs like obesity and neuroblastoma. Future drug development targeting monoaminergic systems could take advantage of monitoring the AP-2β levels during clinical studies for better management and treatment of MNDs.

## Data Availability

Not applicable.

## Abbreviations

CNS: Central nerve system
PNS: Prepheral nerve system
MNDs: Monoamine neurotransmitter disorders
DA: Dopamine
NA: Noradrenaline
5-HT: Serotonin
T2D: Type 2 diabetes
AD: Alzheimer's disease
TCA: Tricyclic antidepressants
SSRIs: Selective serotonin reuptake inhibitors
SNRIs: Serotonin and norepinephrine reuptake inhibitors
MAOIs: Monoamine oxidase inhibitors
COMTIs: Catechol-*O*-methyltransferase inhibitors
AP-2β: Transcription factor activating protein 2 beta
*5-HTT*: Serotonin transporter
*DBH*: Dopamine-beta-hydroxylase
*VMAT2*: Vesicular monoamine transporter 2
*WWOX*: WW Domain Containing Oxidoreductase
*GREB1*: Growth Regulating Estrogen Receptor Binding 1
*CDH2*: Cadherin-2
*HPSE*: Heparanase
IGSF11: Immunoglobulin Superfamily Member 11
BOFS: Branchio-oculo-facial syndrome
COL2A1: Core promoter of type II collagen
*HMOX1*: Heme Oxygenase 1
IRS-1: Insulin resistance substrate 1
PKC: Protein kinase C
PKA: CAMP-dependent protein kinase A
KCTD1: The potassium channel tetramerization domain 1
KCTD15: Potassium channel tetramerization domain 15
PKD: Protein kinase D
HIF-2α: Hypoxia-inducible factor-2alpha
CITED2: Cpb/p300-interacting transactivator 2
CITED4: Cpb/p300-interacting transactivator 4
YEATS4: YEATS Domain-containing protein 4
MYC: MYC Proto-Oncogene, bHLH transcription factor
TP53: Cellular tumor antigen p53
UBC: Ubiquitin carrier protein 9
UBE2I: Ubiquitin conjugating enzyme E2 I
SYT4: Synaptotagmin-4
*TH*: Tyrosine hydroxylase
*PNMT*: Phenylethanolamine *N*-methyltransferase
*MAO-A*: Monoamine oxidase-A
DRD1_A_: Dopamine receptor D1A
AC: Adenylyl cyclase
cAMP: Cyclic adenosine monophosphate
HAV: Homovanillic acid
MGPG: 3- Methoxy-4-hydroxy-phenylgly
*TPH*: Tryptophan hydroxylase
*AADC*: Aromatic l‐Amino acid decarboxylase
*VMAT2*: Vesicular monoamine transporter 2 gene
IL-6: Interleukin 6
MCP: Monocyte chemoattractant protein-1
TNF-alpha: Tumour necrosis factor-alpha
IRS-I: Insulin receptor substrate 1
GLUT4: Glucose transport 4
apoE: Apolipoprotein E
TFAs: Transcription factor analogues
DMF: Dimethyl fumarate
